# Chemical Compositions, Mosquito Larvicidal and Antimicrobial Activities of Leaf Essential Oils of Eleven Species of Lauraceae from Vietnam

**DOI:** 10.3390/plants9050606

**Published:** 2020-05-10

**Authors:** Dao Thi Minh Chau, Nguyen Thanh Chung, Le Thi Huong, Nguyen Huy Hung, Isiaka A. Ogunwande, Do Ngoc Dai, William N. Setzer

**Affiliations:** 1 Institute of Biochemical Technology and Environment, Vinh University, 182 Le Duan, Vinh City 4300, Nghệ An Province, Vietnam; daochau27@gmail.com; 2 Graduate University of Science and Technology, Vietnam Academy of Science and Technology, 18-Hoang Quoc Viet, Cau Giay 10072, Hanoi, Vietnam; chungphoat@gmail.com; 3 School of Natural Science Education, Vinh University, 182 Le Duan, Vinh City 4300, Nghệ An Province, Vietnam; lehuong223@gmail.com; 4 Center for Advanced Chemistry, Institute of Research and Development, Duy Tan University, 03 Quang Trung, Da Nang 5000, Vietnam; nguyenhuyhung@duytan.edu.vn; 5 Foresight Institute of Research and Translation, University Road, Aleku Area, Osogbo 230271, Nigeria; isiakaogunwande@gmail.com; 6 Faculty of Agriculture, Forestry and Fishery, Nghe An College of Economics, 51-Ly Tu Trong, Vinh City 4300, Nghe An Province, Vietnam; 7 Department of Chemistry, University of Alabama in Huntsville, Huntsville, AL 35899, USA; 8 Aromatic Plant Research Center, 230 N 1200 E, Suite 100, Lehi, UT 84043, USA

**Keywords:** Beilschmiedia, Cryptocarya, Litsea, Machilus, Neolitsea, Phoebe, Aedes, Culex, antibacterial, antifungal

## Abstract

The Lauraceae is a family rich in aromatic and medicinal plants. Likewise, essential oils derived from members of this family have demonstrated a myriad of biological activities. It is hypothesized that members of the Lauraceae from Vietnam will yield essential oils that may be useful in controlling mosquito populations and treating microbial infections. In this work, the leaf essential oils of eleven species of Lauraceae (*Beilschmiedia erythrophloia*, *B. robusta*, *B. yunnanensis*, *Cryptocarya concinna*, *C. impressa*, *C. infectoria*, *Litsea viridis*, *Machilus balansa*, *M. grandifolia*, *Neolitsea ellipsoidea*, and *Phoebe angustifolia*) have been obtained by hydrodistillation and the chemical compositions analyzed by gas chromatography – mass spectrometry (GC-MS) and gas chromatography with flame ionization detection (GC-FID). The essential oils were screened for larvicidal activity against *Aedes aegypti*, *Ae. albopictus*, and *Culex quinquefasciatus*, and for antimicrobial activity against *Enterococcus faecalis*, *Staphylococcus aureus*, *Bacillus cereus*, *Escherichia coli*, *Pseudomonas aeruginosa*, *Salmonella enterica*, and *Candida albicans*. The leaf essential oil of *N. ellipsoidea*, rich in (*E*)-β-ocimene (87.6%), showed excellent larvicidal activity against *Ae. aegypti* with a 24 h LC_50_ of 6.59 μg/mL. The leaf essential oil of *C. infectoria*, dominated by germacrene D (55.5%) and bicyclogermacrene (11.4%), exhibited remarkable larvicidal activity against *Cx. quinquefasciatus* (48 h LC_50_ = 0.40 μg/mL). *N. ellipsoidea* leaf essential oil also demonstrated notable antibacterial activity against *E. faecalis* and *B. cereus* with minimum inhibitory concentration (MIC) values of 16 μg/mL, while the leaf essential oil of *C. impressa* showed excellent anticandidal with an MIC of 16 μg/mL. Leaf essential oils from the Lauraceae should be considered for utilization as alternative agents for controlling mosquito populations and as antimicrobial agents.

## 1. Introduction

The Lauraceae is made up of around 55 genera and 3000 species of tropical and warm temperate trees and shrubs, with Southeast Asia and Brazil serving as species-rich hot spots [1]. Several members of the family are commercially important, including the avocado (*Persea americana* Mill.) for its fruit, bay leaf (*Laurus nobilis* L.) used in cooking, and the spice cinnamon (*Cinnamomum verum* J. Presl) [2]. Several Lauraceae species have been used medicinally, including sassafras (*Sassafras albidum* (Nutt.) Nees) [3] and spicebush (*Lindera benzoin* (L.) Blume) [4]. Many species of Lauraceae contain essential oils that have found use in the flavor and fragrance industry [5], e.g., Brazilian rosewood (*Aniba rosaeodora* Ducke) [6], camphor tree, ravintsara, ho leaf (*Cinnamomum camphora* (L.) J. Presl.) [7], and aromatic litsea (*Litsea cubeba* (Lour.) Pers.) [8].

Based on the utility and properties of Lauraceae essential oils, it is hypothesized that members of the Lauraceae found in Vietnam have biologically active essential oils that may be useful in controlling mosquito populations or as antimicrobial agents. Eleven species of Lauraceae from north-central Vietnam have been collected, the essential oils obtained by hydrodistillation, chemical compositions analyzed, and the oils screened for mosquito larvicidal activity and for antimicrobial activity.

The genus *Beilschmiedia* Nees is comprised of around 250 species of trees and shrubs [9] and are widespread in tropical Africa, Madagascar, Asia, Southeast Asia, Melanesia, Australia, New Zealand, North America, Central America, South America, and the Caribbean [10]. The phytochemistry and bioactivity of *Beilschmiedia* has been reviewed [11].

*Beilschmiedia erythrophloia* Hayata is a tree found in Taiwan, southern China, Hainan Island, and Ryukyu Islands (Japan) [12,13]. In Vietnam, the tree is found in Nghệ An, Hà Tĩnh, and Đồng Nai provinces [14]. Previous phytochemical studies of *B. erythrophloia* have revealed endiandric acid derivatives from the roots [15,16], the cytotoxic alkaloid beischamide from the stems [13], and a leaf essential oil rich in (*E*)-caryophyllene and α-humulene [17].

*Beilschmiedia robusta* C.K. Allen is a tree, 10–15 m tall that is recorded from Guangzi, southwestern Guizhou, Xizang, and Yunnan provinces in China [12,18]. In Vietnam, the tree is found in Lào Cai, Ninh Bình, and Nghệ An provinces [14]. A perusal of the literature has revealed that there have been no previous phytochemical investigations of *B. robusta*.

*Beilschmiedia yunnanensis* H.H. Hu is a tree, up to 18 m tall and is found in Guangdong, southern Guangxi, and southern Yunnan provinces in China [12]. In Vietnam, the tree is found in Lào Cai, Nghệ An, and Hà Tĩnh provinces [14]. A literature search has revealed that there have been no previous phytochemical investigations of *B. yunnanensis*.

*Cryptocarya* R. Br. is a pantropical genus of around 300 species [19]. *Cryptocarya concinna* Hance (syn. *Cryptocarya konishii* Hayata, *Cryptocarya lenticellata* Lecomte, *Cryptocarya microcarpa* F.N. Wei) is a tree up to 18 m tall, and ranges from southern China (Guangdong, Guangxi, southeastern Guizhou, Hainan, Jiangxi, and Taiwan) to northern Vietnam [9,12]. In Vietnam, it has been recorded in Hà Giang, Tuyên Quang, Cao Bằng, Vĩnh Phúc, Hải Phòng, Thanh Hóa, Nghệ An, Hà Tĩnh, Thừa Thiên Huế provinces [14]. Previous investigations of the phytochemistry of *C. concinna* have shown the roots to contain cytotoxic cryptocaryone [20], the leaves to contain cytotoxic cryptoconcatones K and L [21], and the wood to contain cytotoxic cryptocaryone and kurzichalcolactone A and antifungal cryptocaryanone A and kurzichalcolactone B [22]. There have been no previous reports on essential oils from *C. concinna*.

*Cryptocarya impressa* Miq. is native to Vietnam, Laos, the Malay Peninsula, Borneo and Sumatra [23]. In Vietnam, the plant has been recorded in Hòa Bình, Hà Nội, Hải Dương, Ninh Bình, Nghệ An, and Gia Lai provinces [14]. To our knowledge, there have been no reports on the phytochemistry of *C. impressa*.

*Cryptocarya infectoria* (Blume) Miq. (syn. *Cylicodaphne infectoria* Blume) is a tree up to 33 m tall that is native to Indo-China and Malesia [24,25,26]. In Vietnam, this tree is found in Lào Cai, Phú Thọ, Vĩnh Phúc, Thanh Hoá, Nghệ An, Hà Tĩnh, and Thừa Thiên Huế provinces [14]. The cytotoxic dihydrochalcones, cryptocaryone and infectocaryone, and the flavonoids cryptocaryanones A and B have been isolated from the methanol bark extract of *C. infectoria* [27,28]. The isoquinoline alkaloids atherosperminine, *N*-methylisococlaurine, and *N*-methyllaurotetanine have also been isolated from the bark of *C. infectoria* [29]. There have been apparently no essential oil analyses on this plant, however.

The genus *Litsea* Lam. consists of around 300 species distributed in tropical and warm subtropical regions of Asia, Australia, and the Americas [19]. *Litsea viridis* H. Liu is a small tree, 3-6 m tall, found in south-eastern Yunnan province (China) and Cao Bằng, Nghệ An, Đà Nẵng, and Đắk Lắk provinces (Vietnam) [12,14]. There do not seem to be any previous studies on the phytochemistry of this plant.

The genus *Machilus* Rumph. ex Nees is comprised of around 100 species distributed in southern and south-eastern Asia [12,14]. *Machilus balansae* (Airy Shaw) F.N. Wei & S.C. Tang (syn. *Persea balansae* Airy Shaw) is endemic to Vietnam and is generally found at low elevations in north Vietnam [30]. *Machilus grandifolia* S.K. Lee & F.N. Wei is now regarded as a new synonym of *M. balansae* [30]. To our knowledge, there have been no phytochemical studies reported on *M. balansae* or *M. grandifolia*.

The genus *Neolitsea* (Benth.) Merr. Contains around 85 species distributed from Indo-Malaysia to East Asia [12,14]. *Neolitsea ellipsoidea* K.C. Allen is a tree up to 30 m in height [31]. The species has been recorded in Hainan (China) and Vietnam (Hoà Bình, Quảng Ninh, Hà Tĩnh, and Gia Lai provinces). To our knowledge there have been no reports on the phytochemistry of this species.

There are around 100 species in the genus *Phoebe* Nees [19], which range from the Neotropics (Mexico, south to Brazil, Bolivia, and Argentina) and Southeast Asia (southern China, Vietnam, Thailand, Myanmar, Cambodia, and Singapore), as well as Indonesia, New Guinea, and India [9].

*Phoebe angustifolia* Meisn. is a small shrub found in southeastern Yunnan (China), Myanmar, India, and Vietnam [12]. In Vietnam, the species has been recorded in Thanh Hóa, Nghệ An, Thừa Thiên Huế, and Quảng Nam provinces [14]. The leaf essential oil of *P. angustifolia* from Vietnam has been reported, which showed the major components to be spathulenol (17.0%), palmitic acid (13.0%), sabinene (6.4%), bicyclogermacrene (5.9%), and artemisia triene (5.1%) [32].

## 2. Results and Discussion

The essential oil collection details and yields are summarized in Table 1.

### 2.1. Essential Oil Compositions

The essential oil compositions of *B. erythrophloia*, *B. robusta*, and *B. yunnanensis* are compiled in Table 2. All three of the *Beilschmiedia* leaf essential oils were dominated by sesquiterpene hydrocarbons. A preponderance of sesquiterpene hydrocarbons has been previously seen in *Beilschmiedia* leaf essential oils from Malaysia [33] and from Costa Rica [34].

The major components in *B. erythrophloia* essential oil were bicyclogermacrene (30.5%), (*Z*)-β-ocimene (26.1%), and (*E*)-caryophyllene (18.3%). While qualitatively similar, there are notable differences between the essential oil from Vietnam in this work and that reported by Su and Ho from Taiwan [17]; the sample from Taiwan was rich in α-humulene (21.9%) compared to that from Vietnam (only 2.6%), but poor in bicyclogermacrene (1.2%) compared to that from Vietnam.

Both *B. robusta* and *B. yunnanensis* leaf oils were rich in (*E*)-caryophyllene (22.5% and 16.2%, respectively), α-humulene (13.4% and 9.9%), and bicyclogermacrene (8.6% and 8.4%). The leaf oil of *B. robusta* had a high concentration of germacrene D (20.3%), while *B. yunnanensis* oil was rich in 9-*epi*-(*E*)-caryophyllene (21.2%).

The leaf essential compositions of *C. concinna* (from two locations), *C. impressa*, and *C. infectoria* are listed in Table 3. Sesquiterpene hydrocarbons were abundant in both *C. impressa* and *C. infectoria* leaf essential oils, while oxygenated sesquiterpenoids were abundant in *C. concinna* essential oil from Nam Dong and monoterpene hydrocarbons dominated the leaf oil of *C. concinna* from Pu Hoat.

The leaf essential oils of *C. concinna* from two different collection sites were qualitatively similar, but quantitatively different. That is, the abundant components in the Nam Dong sample were also observed in the Pu Hoat sample, and vice versa. Thus, for example, α-pinene, β-pinene, and myrcene were abundant in the Pu Hoat sample (26.7%, 31.3%, and 11.1%, respectively) but were found in lower concentrations in the sample from Nam Dong (8.2%, 9.0%, and 3.9%). Conversely, the sesquiterpenoids, (*E*)-caryophyllene, spathulenol, and caryophyllene oxide were abundant in the sample from Nam Dong (12.2%, 12.3%, and 21.2%, respectively), but less concentrated in the Pu Hoat sample (5.3%, 1.1%, and 0.4%).

The major components of the leaf essential oil of *C. impressa* were bicyclogermacrene (18.7%), (*E*)-caryophyllene (10.8%), dodecanal (10.8%), (*E*,*E*)-α-farnesene (7.9%), and α-humulene (6.3%). Germacrene D (55.5%) dominated the essential oil composition of *C. infectoria*, which was also composed of bicyclogermacrene (11.4%) and δ-elemene (5.1%) as major components. 

The chemical compositions of the leaf essential oils of *L. viridis*, *M. balansae*, *M. grandifolia*, *N. ellisoidea*, and *P. angustifolia* are compiled in Table 4.

The major components in *L. viridis* leaf essential oil were bicyclogermacrene (25.5%), decanal (14.4%), α-pinene (11.1%), and β-pinene (8.3%). This is the first report on the essential oil from this plant.

Although *M. balansae* and *M. grandifolia* are considered conspecific, the essential oil compositions showed pronounced differences. The leaf oil of *M. balansae* was dominated by bicyclogermacrene (41.5%), which was not detected in the essential oil of *M. grandifolia*. Likewise, the sesquiterpene alcohols (*E*)-nerolidol and globulol were abundant constituents in *M. grandifolia* (22.7% and 10.2%, respectively), but (*E*)-nerolidol was much lower in *M. balansae* (8.7%) and globulol was not detected. The taxonomy of these two plants deserves closer scrutiny.

The leaf essential oil of *N. ellipsoidea* was dominated by (*E*)-β-ocimene (87.6%). (*E*)-β-Ocimene was also found to be the dominant compound (85.6%) in the leaf essential oil of *N. polycarpa* from Vietnam [32], and one of the major components in the leaf essential oils of *N. sericea* from Korea (13.3%) [38], *N. variabillima* from Taiwan (13.4%) [39], and *N. aciculata* from Korea (9.7%) [40]. In contrast, (*E*)-β-ocimene was only a minor component in the leaf oils of *N. australiensis*, *N. brassii*, or *N. dealbata* from Australia [41], and *N. pallens* from India [42], and was not observed in *N. foliosa* leaf essential oil from India [43].

The leaf essential oil of *P. angustifolia* from Pù Hoạt Nature Reserve (northern Vietnam) in this study was rich in α-pinene (26.9%), β-pinene (20.8%), spathulenol (5.4%), (*E*)-caryophyllene (5.3%), and *p*-cymene (5.0%), which differs markedly from a previous study on the leaf essential oil from Sao La Nature Reserve (central Vietnam). The previous work reported spathulenol (17.0%), palmitic acid (13.0%), sabinene (6.4%), bicyclogermacrene (5.9%), and artemisia triene (5.1%) to be the major components [32]. There is apparently much variation in the volatile components of this plant.

### 2.2. Larvicidal Activity

The 24-h and 48-h larvicidal activities of Lauraceae leaf essential oils from Vietnam are summarized in Table 5 and Table 6. Note that several essential oils were not tested due to lack of sufficient essential oil.

Of the Lauraceae essential oils screened for larvicidal activity, *N. ellipsoidea* showed the greatest activity against *Ae. aegypti* with 24-h and 48-h LC_50_ values of 6.59 and 4.04 μg/mL, respectively. Similar larvicidal activities were observed against *Cx. quinquefasciatus* (24-h and 48-h LC_50_ = 7.47 and 4.65 μg/mL) for this essential oil. Interestingly, although the larvicidal activities of *C. infectoria* leaf essential oil were not as impressive against *Ae. aegypti* or *Ae. albopictus*, the essential oil did show much better activity against *Cx. quinquefasciatus* (24-h LC_50_ = 10.8 μg/mL), particularly after 48 h of exposure (48-h LC_50_ = 0.402 μg/mL). Unfortunately, the limited quantities available for several of the essential oils precluded larvicidal screening. However, the larvicidal activity of the untested essential oils will be investigated in future studies.

The major component of *N. ellipsoidea* leaf essential oil, (*E*)-β-ocimene (87.6%), is not likely responsible for the observed larvicidal activity. The (*E*)-β-ocimene-rich (94.8%) essential oil of *Porophyllum ruderale* showed an LC_50_ of 173.7 μg/mL against *Ae. aegypti* [44]. Likewise, the essential oil of *Syzygium jambolana*, with (*Z*)-β-ocimene (27.2%) and (*E*)-β-ocimene (12.2%), was inactive against *Ae. aegypti* (LC_50_ = 433 μg/mL) [45]. The excellent larvicidal activity of *N. ellipsoidea* essential oil can likely be attributed to synergistic effects involving minor components.

The leaf essential oil of *C. infectoria* was rich in the germacrene sesquiterpenes germacrene D (55.5%) and bicyclogermacrene (11.4%), and these compounds may be responsible for the larvicidal activity. Germacrene D has demonstrated notable larvicidal activity against *Ae. aegypti* and *Cx. quinquefasciatus* (LC_50_ = 18.8 and 21.3 μg/mL, respectively) [46], and bicyclogermacrene was larvicidal against *Ae. albopictus* and *Cx. tritaeniorhynchus* (LC_50_ = 11.1 and 12.5 μg/mL, respectively) [47].

The marginal larvicidal activity of *C. concinna* from Nam Dong is consistent with the marginal activities observed for the major components. (*E*)-Caryophyllene, caryophyllene oxide, and α-pinene have shown modest mosquito larvicidal activities [48]. β-Pinene, however, has been shown to be more active than α-pinene: (–)-β-pinene (LC_50_ = 65 μg/mL against *Cx. quinquefasciatus*) [49], (LC_50_ = 15.4 μg/mL against *Ae. aegypti*) [50]; (+)-β-pinene (LC_50_ = 22.4 μg/mL against *Ae. aegypti*) [49]. Spathulenol-rich essential oils have also shown only marginal larvicidal activities. The stem essential oil of *Tephrosia toxicaria* (42.3% spathulenol) had an LC_50_ of 63.1 μg/mL against *Ae. aegypti* [51], while *Guarea sylvatica* essential oil from branches (14.3% spathulenol) showed LC_50_ against *Ae. aegypti* of 274 μg/mL [52].

### 2.3. Antimicrobial Activity

Several of the leaf essential oils of the Lauraceae were screened for antimicrobial activity (Table 7). All of the essential oils tested showed good antibacterial activities against the Gram-positive organisms. Both *L. viridis* and *N. ellipsoidea* leaf essential oils demonstrated particularly notable activities against *E. faecalis* and *B. cereus* with minimum inhibitory concentration (MIC) values of 16 μg/mL. The leaf essential oil of *C. impressa* also showed excellent anticandidal activity against *C. albicans* with an MIC of 16 μg/mL.

The major component of *L. viridis* leaf oil, bicyclogermacrene, has shown antibacterial activity against *B. cereus* [53]. Likewise, β-pinene was shown to be active against *E. faecalis* [54] as well as several other Gram-positive organisms [55]. Similarly, α-pinene has activity against several Gram-positive bacteria [55,56]. Decanal has also exhibited antibacterial activity [57,58]. Thus, the major components of *L. viridis* leaf essential oil, bicyclogermacrene, decanal, α-pinene, and β-pinene, can account for the observed antibacterial activity.

(*E*)-β-Ocimene dominated the leaf essential oil of *N. ellipsoidea*, but this compound has demonstrated relatively marginal antibacterial activity [55]. Synergistic interactions of (*E*)-β-ocimene with minor essential oil components may play a role in the antibacterial activity of *N. ellipsoidea* leaf oil.

The components responsible for the anticandidal activity of *C. impressa* leaf essential oil are not obvious. Neither (*E*)-caryophyllene nor α-humulene have shown strong anti-*Candida albicans* activity [54,56]. The anticandidal activity of bicyclogermacrene itself has apparently not been determined. However, essential oils rich in both bicyclogermacrene and (*E*)-caryophyllene do not exhibit notable activity against *Candida* spp. [59,60]. Dodecanal, however, has shown activity against *C. albicans* with an MIC of 125 μg/mL [61].

## 3. Materials and Methods 

### 3.1. Plant Collection

Leaves were collected from wild-growing trees in north-central Vietnam. Plants were identified by Do Ngoc Dai and voucher specimens (Table 1) have been deposited in the plant specimen room, Faculty Agriculture, Forestry and Fishery, Nghe An, College of Economics. In each case, the fresh leaves were chopped and 2.0 kg was subjected to hydrodistillation using a Clevenger-type apparatus.

### 3.2. Analysis of the Oils

Gas chromatographic (GC) analysis was performed on an Agilent Technologies HP 7890A Plus Gas chromatograph equipped with a FID and fitted with HP-5ms column (30 m × 0.25 mm, film thickness 0.25 μm, Agilent Technologies, Santa Clara, CA, USA). The analytical conditions were: carrier gas H_2_ (1 mL/min), injector temperature (PTV: programmable temperature vaporization) 250 °C, detector temperature 260 °C, column temperature programmed from 60 °C (2 min hold) to 220 °C (10 min hold) at 4 °C/min. Samples were injected using a split mode with a split ratio of 10:1. The volume injected was 1.0 μL. Inlet pressure was 6.1 kPa.

An Agilent Technologies (Santa Clara, CA, USA) HP 7890A Plus Chromatograph fitted with a fused silica capillary HP-5ms column (30 m × 0.25 mm, film thickness 0.25 μm) and interfaced with a mass spectrometer HP 5973 MSD was used for the GC/MS analysis, under the same conditions as those used for GC analysis. The conditions were the same as described above with He (1 mL/min) as carrier gas. The MS conditions were as follows: ionization voltage 70 eV; emission current 40 mA; acquisitions scan mass range of 35–350 amu at a sampling rate of 1.0 scan/s. Compound identification was carried out by comparison of the MS fragmentation patterns and calculated retention indices with those available in the databases [35,36,37] and, when available, with standard substances.

### 3.3. Mosquito Larvicidal Assays

Larvicidal activities against *Aedes aegypti*, *Aedes albopictus*, and *Culex quinquefasciatus* were carried out as previously described [62]; LC_50_ values, LC_90_ values, and 95% confidence limits were determined by log-probit analysis using Minitab^®^ 19 (Minitab, LLC, State College, PA, USA).

### 3.4. Antimicrobial Assays

The bacterial growth inhibition of the essential oils was evaluated using three strains of Gram-positive test bacteria, *Enterococcus faecalis* (ATCC299212), *Staphylococcus aureus* (ATCC25923), *Bacillus cereus* (ATCC14579), three strains of Gram-negative test bacteria, *Escherichia coli* (ATCC 25922), *Pseudomonas aeruginosa* (ATCC27853), *Salmonella enterica* (ATCC13076) and one strain of yeast, *Candida albicans* (ATCC 10231). Minimum inhibitory concentration (MIC) and median inhibitory concentration (IC_50_) values were measured by the microdilution broth susceptibility assay as previously described [62]. 

## 4. Conclusions

Of the eleven species of Lauraceae examined in this work, the leaf essential oil of *Neolitsea ellipsoidea*, dominated by (*E*)-β-ocimene, showed excellent larvicidal activity against *Aedes aegypti* and antibacterial activity against *Enterococcus faecalis* and *Bacillus cereus*; *Cryptocarya infectoria* leaf essential oil, rich in germacrene D and bicyclogermacrene, showed excellent larvicidal activity on *Culex quinquefasciatus* and anticandidal activity against *Candida albicans*. The leaf essential oil of *Litsea viridis*, which was rich in bicyclogermacrene, also showed good antibacterial properties. The biological properties of these Lauraceae essential oils suggest that they may serve as potential “green” alternatives, as also described for Lamiaceae family plants [63], for use as insect control or antimicrobial agents.

## Figures and Tables

**Table 1:** Plant collection and hydrodistillation details of Lauraceae from Vietnam.

Plant Species	Vietnamese Name	Collection Site	Voucher Number	Collection Month/Year	Yield (%, v/w)
*Beilschmiedia erythrophloia* Hayata	Chắp, Kết gỗ đỏ	Pù Hoạt Nature Reserve; 19°41′40″ N, 104°49′31″ E, elev, 678 m	803	7/2019	0.12
*Beilschmiedia robusta* C.K. Allen	Chắp to khỏ, Két to khỏe	Pù Hoạt Nature Reserve; 19°41′37″ N, 104°49′30″ E, elev. 677 m	827	9/2019	0.14
*Beilschmiedia yunnanensis* H.H. Hu	Chắp vân nam, Két vân nam, Mong vân nam	Vũ Quang National park; 18°17′15″ N, 105°21′39″ E, elev. 153 m	799	7/2019	0.15
*Cryptocarya concinna* Hance	Ẩn hạch quả vàng, Mò quả vàng, Kháo	Nam Đông District, Thừa Thiên Huế Province; 16°13′9″ N, 107°43′28″ E, elev. 124 m	791	7/2019	0.33
		Pù Hoạt Nature Reserve; 19°42′18″ N, 104°49′42″ E, elev. 648 m	801	7/2019	0.36
*Cryptocarya impressa* Miq. Syn.: *Cryptocarya venosa* Meisn. ex Hook.f.	Mò quả to, Mò quả xanh, Ẩn hạch quả to	Pù Hoạt Nature Reserve; 19°42′18″ N, 104°49′42″ E, elev. 648 m	826	9/2019	0.22
*Cryptocarya infectoria* (Blume) Miq. Syn.: *Caryodaphne infectoria* Blume	Cà đuối nhuộm, Ẩn hạch nhuộm, Cà đuối tai nghé	Pù Hoạt Nature Reserve; 19°42′18″ N, 104°49′42″ E, elev. 648 m	767	4/2019	0.25
*Litsea viridis* H. Liou	Bời lời xanh	Pù Hoạt Nature Reserve; 19°42′18″ N, 104°49′42″ E, elev. 648 m	806	8/2019	0.21
*Machilus balansa* (Airy Shaw) F.N. Wei & S.C. Tang Syn.: *Persea balansae* Airy Shaw	Kháo balansa, Rè balansa	Pù Mát National Park; 18°58′14″ N, 104°48′2″ E, elev. 376 m	828	9/2019	0.42
*Machilus grandifolia* S.K. Lee & F.N. Wei	Kháo lá to	Nam Đông District, Thừa Thiên Huế Province; 16°13′9″ N, 107°43′28″ E, elev. ZZ m	779	7/2019	0.18
*Neolitsea ellipsoidea* K.C. Allen	Nô bầu dục, Bài nhài lá bầu dục, Tam tầng	Vũ Quang National park; 18°17′15″ N, 105°21′39″ E, elev. 124 m	802	7/2019	0.31
*Phoebe angustifolia* Meisn. Syn.: *Phoebe angustifolia* var. *annamensis* Liou	Re trắng lá hẹp, Sụ lá hẹp, Dù dà mò cát	Pù Hoạt Nature Reserve; 19°49′7″ N, 104°55′38″ E, elev. 465 m	785	7/2019	0.45

**Table 2:** Chemical compositions of the leaf essential oils of *Beilschmiedia* species collected in Vietnam.

RI_calc_	RI_db_	Compounds	Percent Composition		
			*B.e.*	*B.r.*	*B.y.*
930	924	α-Thujene	-	0.1	0.6
939	932	α-Pinene	3.2	2.9	6.0
955	946	Camphene	0.2	0.2	0.2
979	969	Sabinene	0.1	0.6	1.9
984	974	β-Pinene	0.6	2.7	4.7
992	988	Myrcene	0.5	0.4	0.8
1010	1002	α-Phellandrene	0.1	-	0.1
1022	1014	α-Terpinene	-	0.5	1.5
1030	1020	*p*-Cymene	-	0.3	0.8
1034	1024	Limonene	0.2	0.8	1.2
1035	1025	β-Phellandrene	-	0.1	0.6
1039	1032	(*Z*)-β-Ocimene	26.1	-	0.1
1049	1044	(*E*)-β-Ocimene	3.6	0.5	-
1063	1054	γ-Terpinene	-	0.9	2.6
1094	1086	Terpinolene	-	0.4	0.8
1117	1113	(*E*)-4,8-Dimethylnona-1,3,7-triene	-	-	0.2
1131	1128	*allo*-Ocimene	0.6	-	-
1188	1174	Terpinen-4-ol	-	0.5	1.8
1200	1186	α-Terpineol	-	-	0.2
1294	1287	Bornyl acetate	0.3	-	-
1348	1335	δ-Elemene	1.5	0.3	0.3
1360	1345	α-Cubebene	-	0.3	-
1365	1359	Neryl acetate	-	-	0.2
1384	1373	α-Ylangene	-	0.1	-
1386	1387	β-Cubebene	-	-	0.1
1389	1374	α-Copaene	0.3	0.7	0.2
1397	1390	7-*epi*-Sesquithujene	0.5	0.7	1.0
1399	1387	β-Bourbonene	-	0.9	-
1404	1389	β-Elemene	1.0	1.0	0.6
1425	1411	*cis*-α-Bergamotene	-	0.4	0.4
1428	1409	α-Gurjunene	-	-	0.3
1437	1417	(*E*)-Caryophyllene	18.3	22.5	16.2
1446	1432	*trans*-α-bergamotene	0.5	1.2	1.1
1452	1437	α-Guaiene	-	0.4	0.4
1457	1439	Aromadendrene	0.7	1.5	1.8
1460	1440	(*Z*)-β-Farnesene	0.3	0.2	0.5
1466	1448	*cis*-Muurola-3,5-diene	-	0.2	-
1471	1452	α-Humulene	2.6	13.4	9.9
1479	1464	9-*epi*-(*E*)-Caryophyllene	0.4	0.5	21.2
1488	1481	γ-Curcumene	-	-	0.2
1490	1478	γ-Muurolene	0.1	1.9	0.3
1494	1483	α-Amorphene	-	0.6	-
1498	1484	Germacrene D	2.7	20.3	1.1
1504	1489	β-Selinene	-	-	0.2
1505	1492	δ-Selinene	-	0.4	0.2
1507	1490	9-Aromadendrene	-	-	0.9
1512	1505	(*E*,*E*)-α-Farnesene	-	1.4	-
1512	1496	Viridiflorene	-	2.4	2.0
1514	1500	Bicyclogermacrene	30.5	8.6	8.4
1520	1514	β-Curcumene	-	-	0.2
1521	1511	δ-Amorphene	0.1	-	-
1522	1509	α-Bulnesene	-	-	0.3
1530	1513	γ-Cadinene	0.1	0.8	0.2
1537	1522	δ-Cadinene	0.5	2.9	0.5
1540	1528	Zonarene	-	0.2	-
1547	1533	*trans*-Cadina-1,4-diene	-	0.2	-
1552	1537	α-Cadinene	-	0.2	-
1562	1548	Elemol	0.2	-	-
1571	1561	(*E*)-Nerolidol	-	0.2	1.4
1577	1559	Germacrene B	0.2	-	-
1588	1567	Palustrol	-	-	0.4
1599	1577	Spathulenol	0.9	0.6	1.0
1604	1592	Viridiflorol	-	0.4	1.2
1605	1582	Caryophyllene oxide	0.6	0.4	-
1612	1595	Cubeban-11-ol	-	0.6	-
1615	1600	Guaiol	0.3	-	1.0
1621	1600	Rosifoliol	-	0.2	0.3
1625	1602	Ledol	-	-	1.1
1632	1608	Humulene epoxide II	-	0.2	0.2
1642	1637	5-Guaiene-11-ol	-	-	0.2
1658	1640	*epi*-α-Muurolol	-	0.2	-
1659	1638	*epi*-α-Cadinol	-	0.2	-
1670	1652	α-Eudesmol	0.1	-	-
1673	1652	α-Cadinol	0.1	0.5	-
1674	1662	7-*epi*-α-Eudesmol	0.3	-	-
1683	1670	*epi*-β-Bisabolol	-	-	0.1
1759	1732	Zerumbone	0.1	-	-
		Monoterpene hydrocarbons	35.2	10.4	21.9
		Oxygenated monoterpenoids	0.3	0.5	2.2
		Sesquiterpene hydrocarbons	60.3	84.2	68.5
		Oxygenated sesquiterpenoids	2.6	3.5	6.9
		Others	0.0	0.0	0.2
		Total identified	98.4	98.6	99.7

RI_calc_ = Retention index determined with respect to a homologous series of *n*-alkanes on a HP-5ms column, RI_db_ = Retention index from the databases [35,36,37], *B.e.* = *Beilschmiedia erythrophloia*, *B.r.* = *Beilschmiedia robusta*, *B.y.* = *Beilschmiedia yunnanensis*.

**Table 3:** Chemical compositions of the leaf essential oils of *Cryptocarya* species collected in Vietnam.

RI_calc_	RI_db_	Compound	Percent Composition			
			*C.c.* N.D.	*C.c.* P.H.	*C.im.*	*C.in.*
930	927	α-thujene	tr	0.1	-	-
931	932	α-Pinene	8.2	26.7	4.1	0.8
945	948	α-Fenchene	tr	-	-	-
955	953	Camphene	0.2	0.4	0.3	0.6
967	961	Benzaldehyde	-	-	-	0.1
970	971	Sabinene	tr	-	-	-
975	978	β-Pinene	9.0	31.3	2.7	0.2
986	989	Myrcene	3.9	11.1	3.9	-
1010	1002	α-Phellandrene	-	-	2.5	-
1012	1009	δ-3-Carene	0.1	-	0.2	-
1027	1025	*p*-Cymene	0.1	-	0.6	-
1027	1030	Limonene	2.0	2.8	0.9	0.2
1028	1031	β-Phellandrene	tr	0.3	-	-
1033	1034	(*Z*)-β-Ocimene	tr	0.6	0.3	-
1043	1046	(*E*)-β-Ocimene	0.2	8.8	4.0	-
1063	1054	γ-Terpinene	-	0.1	-	-
1094	1086	Terpinolene	-	0.1	0.4	-
1096	1098	Perillene	0.1	-	-	-
1098	1101	α-Pinene oxide	0.2	-	-	-
1101	1095	Linalool	-	1.1	-	3.4
1117	1116	(*E*)-4,8-Dimethylnona-1,3,7-triene	-	-	0.7	-
1137	1139	Nopinone	0.1	-	-	-
1139	1141	*trans*-Pinocarveol	0.3	-	-	-
1144	1145	*trans*-Verbenol	0.1	-	-	-
1161	1164	Pinocarvone	0.1	-	-	-
1194	1195	Myrtenol	0.3	-	-	-
1206	1201	Decanal	-	-	1.6	-
1299	1300	Tridecane	-	-	0.2	-
1308	1305	Undecanal	-	-	0.2	-
1332	1335	δ-Elemene	0.9	0.2	0.7	5.1
1344	1348	α-Cubebene	0.2	-	0.1	0.3
1366	1371	α-Ylangene	0.4	-	-	-
1367	1356	Eugenol	-	-	-	0.1
1373	1375	α-Copaene	0.5	-	0.5	0.8
1381	1382	β-Bourbonene	0.2	-	-	0.3
1384	1373	α-Ylangene	-	-	-	0.4
1385	1387	β-Cubebene	0.1	-	-	-
1387	1390	β-Elemene	0.9	0.1	1.2	2.1
1412	1408	Dodecanal	-	-	10.8	-
1417	1417	(*E*)-Caryophyllene	12.2	5.3	10.8	1.7
1419	1421	(*E*)-α-Ionone	tr	-	-	-
1424	1430	γ-Maaliene	0.2	-	-	-
1427	1430	β-Copaene	0.3	-	-	-
1428	1426	α-Gurjunene	-	-	0.4	-
1430	1432	*trans*-α-Bergamotene	1.6	-	0.9	-
1436	1438	Aromadendrene	1.5	0.8	1.8	-
1445	1437	β-Gurjunene	-	-	-	0.8
1449	1455	Valerena-4,7(11)-diene	0.1	-	-	-
1453	1454	α-Humulene	1.5	0.6	6.3	1.9
1453	1442	α-Maaliene	-	-	0.2	-
1456	1447	Guaia-6,9-diene	-	-	-	0.6
1457	1458	*allo*-Aromadendrene	0.1	-	-	-
1459	1454	Selina-5,11-diene	-	-	0.2	-
1463	1463	*cis*-Muurola-4(14),5-diene	-	-	0.1	-
1466	1454	*cis*-Muurola-3,5-diene	-	-	-	0.2
1472	1475	γ-Muurolene	1.6	0.4	0.7	1.3
1476	1482	α-Amorphene	0.2	-	0.7	0.7
1478	1480	Germacrene D	0.2	1.3	2.5	55.5
1479	1470	9-*epi*-(*E*)-caryophyllene	-	0.2	0.6	0.3
1480	1477	*trans*-Cadina-1(6),4-diene	-	-	-	0.3
1481	1478	γ-Gurjunene	0.1	-	-	-
1486	1489	β-Selinene	0.5	-	0.5	-
1488	1491	Viridiflorene	0.1	-	-	-
1489	1490	γ-Amorphene	0.3	-	-	-
1493	1497	α-Selinene	0.6	-	-	-
1495	1497	α-Muurolene	0.4	-	-	-
1504	1508	β-Bisabolene	0.2	-	-	-
1505	1497	δ-Selinene	-	-	-	0.7
1509	1496	γ-Amorphene	-	-	-	0.3
1512	1515	Cubebol	0.2	-	-	-
1512	1517	(*E*,*E*)-α-Farnesene	-	-	7.9	-
1514	1511	Bicyclogermacrene	-	-	18.7	11.4
1515	1518	δ-Cadinene	0.7	0.7	1.1	-
1518	1519	*trans*-Calamenene	0.3	-	-	-
1520	1512	γ-Cadinene	1.3	0.4	0.3	-
1521	1515	δ-Amorphene	-	-	0.2	0.7
1534	1538	α-Cadinene	0.2	-	-	0.2
1538	1541	α-Calacorene	0.4	-	-	-
1538	1531	*cis*-Calamene	-	-	-	0.2
1546	1549	α-Elemol	0.1	-	-	0.2
1547	1540	*trans*-Cadina-1,4-diene	-	-	-	0.1
1548	1551	Isocaryphyllene oxide	0.9	-	-	-
1556	1560	Germacrene B	0.1	-	0.7	0.6
1558	1560	(*E*)-Nerolidol	0.2	-	0.7	0.1
1559	1560	β-Calacorene	0.3	-	-	-
1560	1551	Selina-3,7(11)-diene	-	-	0.5	-
1565	1566	1,5-Epoxysalvial-4(14)-ene	0.6	-	-	-
1575	1578	Spathulenol	12.3	1.1	1.4	0.1
1580	1587	Caryophyllene oxide	21.2	0.4	0.4	0.2
1583	1590	Globulol	0.7	-	-	-
1583	1579	Dendrolasin	-	-	0.2	-
1594	1593	Scapanol	-	-	-	0.2
1597	1594	Viridiflorol	0.4	-	0.4	-
1604	1612	5-*epi*-7-*epi*-β-Eudesmol	0.2	-	-	-
1607	1613	Humulene epoxide II	1.5	-	0.3	-
1612	1601	Cubeban-11-ol	-	-	0.4	-
1614	1611	Tetradecanal	-	-	1.0	-
1621	1615	Rosifoliol	-	-	0.4	-
1623	1624	Muurola-4,10(14)-dien-1β-ol	0.2	-	-	-
1625	1629	*iso*-Spathulenol	1.8	-	-	-
1630	1630	Caryophylla-4(12),8(13)-dien-5α-ol	0.9	-	-	-
1634	1636	Caryophylla-4(12),8(13)-dien-5β-ol	0.6	-	-	-
1639	1640	τ-Cadinol	0.2	0.4	-	0.3
1641	1644	τ-Muurolol	0.2	-	-	0.2
1642	1637	5-Guaien-11-ol	-	-	0.2	-
1644	1645	δ-Cadinol	0.4	-	-	-
1646	1635	Muurola-4,10(14)-dien-1β-ol	-	-	-	0.1
1652	1656	β-Eudesmol	0.7	-	-	-
1653	1655	α-Cadinol	0.9	-	0.1	0.4
1654	1661	*cis*-Calamenen-10-ol	0.4	-	-	-
1656	1660	Selin-11-en-4α-ol	0.3	-	-	-
1662	1662	9-Methoxycalamenene	0.4	-	-	-
1668	1666	14-Hydroxy-9-*epi*-(*E*)-Caryophyllene	0.8	-	-	-
1955	1958	Palmitic acid	0.2	-	-	-
2116	2114	Phytol	-	-	0.4	-
		Monoterpene hydrocarbons	23.7	82.3	19.9	1.8
		Oxygenated monoterpenoids	1.1	1.1	0.0	3.4
		Sesquiterpene hydrocarbons	28.1	10.0	57.6	86.5
		Oxygenated sesquiterpenoids	46.1	1.9	4.5	1.8
		Diterpenoids	0.0	0.0	0.4	0.0
		Others	0.3	0.0	14.5	0.2
		Total Identified	99.2	95.3	96.9	93.7

RI_calc_ = Retention index determined with respect to a homologous series of *n*-alkanes on a HP-5ms column, RI_db_ = Retention index from the databases [35,36,37], *C.c.* N.D. = *Cryptocarya concinna* from Nam Dong, *C.c.* P.H. = *Cryptocarya concinna* from Pu Hoat, *C.im.* = *Cryptocarya impressa*, *C.in.* = *Cryptocarya infectoria*, tr = trace.

**Table 4:** Chemical compositions of the leaf essential oils of *Litsea viridis*, *Machilus balansae*, *Machilus grandifolia*, *Neolitsea ellipsoidea*, and *Phoebe angustifolia* collected in Vietnam.

RI_calc_	RI_db_	Compound	Percent Composition				
			*L.v.*	*M.b.*	*M.g.*	*N.e.*	*P.a.*
921	923	Tricyclene	-	-	-	-	0.1
923	927	α-Thujene	-	-	-	-	0.1
934	933	α-Pinene	11.1	4.4	0.3	0.2	26.9
949	948	α-Fenchene	0.1	-	-	-	0.1
950	953	Camphene	0.7	0.3	0.3	-	6.1
971	972	Sabinene	-	-	-	-	0.1
979	978	β-Pinene	8.3	1.2	0.4	0.2	20.8
979	978	1-Octen-3-ol	-	-	0.1	-	-
984	984	6-Methylhept-5-en-2-one	-	-	-	-	0.1
990	991	Myrcene	0.4	0.4	-	0.5	1.5
1008	1006	α-Phellandrene	0.1	-	-	-	0.1
1022	1014	α-Terpinene	0.2	-	-	-	-
1026	1025	*p*-Cymene	0.2	-	1.0	-	5.0
1030	1030	Limonene	1.8	0.4	1.3	0.4	3.1
1030	1031	β-Phellandrene	-	-	0.1	-	0.2
1031	1032	1,8-Cineole	-	-	0.1	-	0.4
1034	1034	(*Z*)-β-Ocimene	0.1	0.1	-	3.7	0.3
1046	1046	(*E*)-β-Ocimene	0.3	4.5	-	87.6	0.1
1063	1054	γ-Terpinene	0.5	-	-	-	-
1069	1069	*cis*-Linalool oxide (furanoid)	-	-	0.4	-	-
1086	1086	*trans*-Linalool oxide (furanoid)	-	-	0.4	-	-
1089	1086	Terpinolene	0.4	-	-	-	tr
1090	1093	*p*-Cymenene	-	-	-	-	0.1
1099	1101	α-Pinene oxide	-	-	-	-	0.1
1100	1101	Linalool	-	-	3.3	1.3	0.1
1105	1100	Nonanal	0.2	0.2	-	-	-
1117	1113	(*E*)-4,8-Dimethylnona-1,3,7-triene	0.4	0.4	-	-	-
1119	1119	*endo*-Fenchol	-	-	0.1	-	0.1
1124	1124	*cis-p*-Menth-2-en-1-ol	-	-	0.1	-	-
1141	1141	*trans*-Pinocarveol	-	-	0.1	-	0.3
1142	1142	*trans-p*-Menth-2-en-1-ol	-	-	0.1	-	-
1143	1140	(*E*)-Myroxide	-	-	-	0.2	-
1145	1145	*trans*-Verbenol	-	-	-	-	0.1
1155	1156	Camphene hydrate	-	-	0.1	-	0.1
1163	1164	Pinocarvone	-	-	-	-	0.1
1172	1173	Borneol	-	-	0.1	-	0.5
1183	1184	Terpinen-4-ol	0.2	-	0.1	-	0.1
1186	1184	(3Z)-Hexenyl butanoate	-	-	-	0.3	-
1188	1187	Cryptone	-	-	0.5	-	-
1196	1195	α-Terpineol	-	-	0.3	-	0.6
1206	1208	Decanal	14.4	-	-	-	0.1
1220	1223	*trans*-Carveol	-	-	0.1	-	-
1245	1246	Carvone	-	-	0.1	-	-
1275	1275	*trans*-Ascaridol glycol	-	-	0.1	-	-
1283	1285	Bornyl acetate	-	-	-	-	1.4
1299	1300	Tridecane	-	0.3	-	-	-
1330	1328	*iso*-Dihydro carvyl acetate	-	-	-	-	0.2
1348	1335	δ-Elemene	-	1.7	-	-	-
1358	1361	Neryl acetate	-	-	-	-	0.1
1360	1345	α-Cubebene	-	0.1	-	-	-
1367	1367	Cyclosativene	-	-	0.2	-	-
1377	1378	Geranyl acetate	-	-	0.1	-	-
1377	1372	*iso*-Ledene	0.3	-	-	-	0.1
1378	1375	α-Copaene	0.6	0.5	1.7	-	0.3
1390	1379	Methyl (*E*)-cinnamate	1.5	-	-	-	-
1395	1390	β-Elemene	1.9	1.0	-	0.3	0.1
1404	1406	α-Gurjunene	-	-	0.1	-	0.2
1412	1412	Dodecanal	2.0	-	-	-	-
1418	1416	*cis*-α-Bergamotene	0.6	-	-	-	0.2
1424	1417	(*E*)-Caryophyllene	0.3	8.5	0.1	0.4	5.3
1425	1430	γ-Maaliene	-	-	-	-	0.1
1428	1422	α-Gurjunene	-	0.5	-	-	-
1434	1432	β-Copaene	-	0.2	-	-	-
1436	1434	β-Gurjunene (= Calarene)	-	0.6	-	-	0.1
1438	1432	*trans*-α-Bergamotene	0.6	0.6	-	-	0.8
1442	1438	α-Maaliene	-	0.3	-	-	0.1
1443	1438	Aromadendrene	3.0	4.5	1.0	0.3	1.8
1444	1445	Selina-5,11-diene	-	-	0.1	-	0.2
1445	1445	*epi*-β-Santalene	-	-	-	-	0.1
1445	1437	γ-Elemene	1.0	-	-	-	-
1453	1453	*cis*-Muurola-3,5-diene	0.2	0.2	-	-	-
1454	1454	α-Humulene	0.9	1.4	-	-	0.6
1455	1452	(*E*)-β-Farnesene	0.3	-	-	-	0.3
1457	1459	β-Santalene	-	-	-	-	0.3
1458	1458	*allo*-Aromadendrene	-	-	0.2	-	0.1
1466	1467	*trans*-Muurola-3,5-diene	-	0.5	-	-	-
1471	1476	γ-Gurjunene	-	-	-	-	0.1
1471	1476	Selina-4,11-diene	-	-	0.4	-	-
1479	1478	γ-Muurolene	0.5	0.8	0.6	0.1	0.1
1479	1474	9-*epi*-(*E*)-caryophyllene	0.8	0.8	-	-	-
1482	1483	*trans*-β-Bergamotene	-	-	-	-	0.2
1485	1482	*ar*-Curcumene	0.4	-	-	-	tr
1486	1482	α-Amorphene	-	0.6	0.1	-	-
1488	1484	γ-Curcumene	0.4	-	-	-	-
1489	1491	Viridiflorene	0.2	-	-	-	1.2
1493	1487	β-Selinene	1.2	0.4	2.7	0.6	0.1
1497	1497	α-Muurolene	-	-	0.5	-	0.1
1498	1501	(*Z*)-α-Bisabolene	-	-	-	-	0.1
1498	1496	Germacrene D	1.0	3.1	-	0.1	-
1503	1497	Bicyclogermacrene	25.5	41.5	-	-	1.3
1503	1497	α-Selinene	-	-	1.3	0.5	-
1505	1500	δ-Selinene	0.9	0.5	-	-	-
1506	1508	β-Bisabolene	-	-	-	-	0.3
1508	1511	(*Z*)-γ-Bisabolene	-	-	-	-	0.1
1512	1511	(*E*,*E*)-α-Farnesene	-	1.8	-	-	-
1517	1512	γ-Cadinene	0.3	0.3	0.5	-	0.2
1520	1519	*trans*-Calamenene	-	-	0.8	-	0.1
1520	1517	β-Curcumene	0.5	-	-	-	-
1521	1516	δ-Amorphene	0.2	0.3	-	-	-
1523	1518	δ-Cadinene	0.9	0.7	0.2	0.2	0.2
1542	1531	(*E*)-γ-Bisabolene	1.0	-	-	-	-
1547	1551	Elemicin	-	-	1.2	-	-
1550	1547	(*E*)-α-Bisabolene	0.4	-	-	-	-
1555	1555	(*Z*)-Dihydronerolidol	-	-	2.4	-	-
1560	1545	Selina-3,7(11)-diene	0.4	-	-	-	-
1564	1561	(*E*)-Nerolidol	1.1	8.7	22.7	-	3.9
1568	1568	Maaliol	-	-	-	-	0.1
1569	1568	Palustrol	-	-	-	-	0.1
1569	1570	(*E*)-Dihydronerolidol	-	-	2.8	-	-
1575	1575	Caryolan-8-ol	-	-	0.8	-	-
1577	1568	Germacrene B	1.3	0.8	-	-	-
1578	1578	Spathulenol	0.9	0.6	-	0.6	5.4
1585	1590	Globulol	-	-	10.2	-	1.7
1589	1587	Caryophyllene oxide	-	-	3.7	0.1	1.5
1596	1594	Viridiflorol	0.4	1.6	0.7	-	0.7
1601	1599	Cubeban-11-ol	-	1.0	0.6	-	0.2
1604	1605	Ledol	-	-	0.7	-	0.1
1609	1613	Humulene epoxide II	-	-	1.5	-	0.1
1611	1609	Rosifoliol	0.4	0.4	0.2	-	0.2
1615	1617	Guaiol	0.8	-	-	-	-
1627	1631	1-*epi*-Cubenol	-	-	0.8	-	-
1632	1629	*iso*-Spathulenol	-	-	-	-	0.4
1642	1637	5-Guaien-11-ol	-	0.5	-	-	-
1646	1645	α-Muurolol (= δ-Cadinol)	-	-	1.5	-	0.1
1647	1643	τ-Cadinol	-	0.2	0.7	-	0.3
1649	1645	τ-Muurolol	-	0.1	1.3	-	0.1
1655	1655	α-Cadinol	0.5	0.4	2.9	-	0.2
1658	1660	Selin-11-en-4α-ol	-	0.3	6.7	-	-
1665	1670	*trans*-Calamenen-10-ol	-	-	1.3	-	-
1671	1665	β-Eudesmol	0.6	-	-	-	-
1674	1676	Mustakone	-	-	0.6	-	-
1674	1670	α-Eudesmol	0.3	-	-	-	-
1683	1672	*epi*-β-Bisabolol	0.2	-	-	-	-
1702	1701	10-*nor*-Calamenen-10-one	-	-	0.4	-	-
		Monoterpene hydrocarbons	24.2	11.3	3.3	92.6	64.5
		Oxygenated monoterpenoids	0.2	0.0	6.2	1.5	4.3
		Sesquiterpene hydrocarbons	45.6	72.2	10.6	2.5	14.8
		Oxygenated sesquiterpenoids	5.2	13.8	62.5	0.7	15.3
		Others	18.5	0.9	1.3	0.3	0.1
		Total Identified	93.7	98.2	84.0	97.6	99.1

RI_calc_ = Retention index determined with respect to a homologous series of *n*-alkanes on a HP-5ms column, RI_db_ = Retention index from the databases [35,36,37], *L.v.* = *Litsea viridis, M.b.* = *Machilus balansae, M.g.* = *Machilus grandifolia, N.e.* = *Neolitsea ellisoidea, P.a.* = *Phoebe angustifolia*.

**Table 5:** Twenty-four-hour larvicidal activities of Lauraceae leaf essential oils from Vietnam.

Lauraceae species	LC_50_	LC_90_	χ^2^	*p*
	*Aedes aegypti*			
*Beilschmiedia erythrophloia*	n.t.	n.t.	---	---
*Beilschmiedia robusta*	24.29 (22.36−26.76)	35.22 (31.70−41.19)	0.1421	0.706
*Beilschmiedia yunnanensis*	n.t.	n.t.	---	---
*Cryptocarya concinna* (Nam Dong)	32.54 (30.21−35.36)	42.94 (39.51−47.91)	0.5537	0.758
*Cryptocarya concinna* (Pu Hoat)	23.01 (20.29−25.83)	40.92 (36.50−47.77)	9.298	0.010
*Cryptocarya impressa*	n.t.	n.t.	---	---
*Cryptocarya infectoria*	21.43 (18.85−24.29)	41.88 (37.16−48.79)	13.58	0.004
*Litsea viridis*	n.t.	n.t.	---	---
*Machilus balansae*	n.t.	n.t.	---	---
*Machilus grandifolia*	20.23 (18.61−21.93)	29.29 (26.85−33.10)	0.001037	0.999
*Neolitsea ellipsoidea*	6.587 (1.478−9.219)	14.00 (10.88−17.71)	0.000224	1.000
*Phoebe angustifolia*	24.29 (22.36−26.76)	35.22 (31.70−41.19)	0.1421	0.931
	*Aedes albopictus*			
*Beilschmiedia erythrophloia*	n.t.	n.t.	---	---
*Beilschmiedia robusta*	n.t.	n.t.	---	---
*Beilschmiedia yunnanensis*	n.t.	n.t.	---	---
*Cryptocarya concinna* (Nam Dong)	34.21 (31.81−37.04)	43.97 (40.67−48.59)	4.651	0.098
*Cryptocarya concinna* (Pu Hoat)	n.t.	n.t.	---	---
*Cryptocarya impressa*	n.t.	n.t.	---	---
*Cryptocarya infectoria*	61.34 (56.76−67.52)	81.29 (73.86−93.08)	3.000	0.223
*Litsea viridis*	n.t.	n.t.	---	---
*Machilus balansae*	n.t.	n.t.	---	---
*Machilus grandifolia*	16.48 (14.82−18.02)	25.00 (22.90−28.16)	1.86 × 10^−5^	1.000
*Neolitsea ellipsoidea*	n.t.	n.t.	---	---
*Phoebe angustifolia*	40.18 (36.12−44.88)	69.56 (62.08−80.81)	31.94	0.000
	*Culex quinquefasciatus*			
*Beilschmiedia erythrophloia*	n.t.	n.t.	---	---
*Beilschmiedia robusta*	n.t.	n.t.	---	---
*Beilschmiedia yunnanensis*	n.t.	n.t.	---	---
*Cryptocarya concinna* (Nam Dong)	56.28 (52.14−62.30)	75.33 (67.95−88.18)	0.5537	0.758
*Cryptocarya concinna* (Pu Hoat)	n.t.	n.t.	---	---
*Cryptocarya impressa*	n.t.	n.t.	---	---
*Cryptocarya infectoria*	10.82 (6.86−14.27)	53.37 (41.49−79.45)	18.66	0.000
*Litsea viridis*	n.t.	n.t.	---	---
*Machilus balansae*	n.t.	n.t.	---	---
*Machilus grandifolia*	13.59 (11.51−15.24)	22.48 (20.34−25.94)	6.1 × 10^−6^	1.000
*Neolitsea ellipsoidea*	7.465 (3.904−9.956)	19.84 (16.52−25.64)	0.1427	0.931
*Phoebe angustifolia*	20.70 (19.36−21.96)	26.60 (25.10−28.63)	0.000	1.000

n.t. = not tested due to insufficient essential oil.

**Table 6:** Forty-eight-hour larvicidal activities of Lauraceae leaf essential oils from Vietnam.

Lauraceae species	LC_50_	LC_90_	χ^2^	*p*
	*Aedes aegypti*			
*Beilschmiedia erythrophloia*	n.t.	n.t.	---	---
*Beilschmiedia robusta*	22.00 (19.81−24.45)	35.64 (31.82−41.93)	0.6316	0.427
*Beilschmiedia yunnanensis*	n.t.	n.t.	---	---
*Cryptocarya concinna* (Nam Dong)	32.03 (29.72−34.84)	42.58 (39.12−47.64)	0.1879	0.910
*Cryptocarya concinna* (Pu Hoat)	16.22 (12.81−18.90)	33.46 (29.37−40.63)	1.028	0.598
*Cryptocarya impressa*	n.t.	n.t.	---	---
*Cryptocarya infectoria*	18.94 (16.39−21.65)	39.12 (34.54−45.97)	13.16	0.004
*Litsea viridis*	n.t.	n.t.	---	---
*Machilus balansae*	n.t.	n.t.	---	---
*Machilus grandifolia*	16.17 (14.61−17.64)	24.03 (22.07−26.93)	1.4 × 10^−6^	1.000
*Neolitsea ellipsoidea*	4.038 (0.004−7.585)	11.12 (2.12−14.74)	0.004798	0.998
*Phoebe angustifolia*	22.46 (20.59−24.69)	33.44 (30.10−39.07)	0.06258	0.969
	*Aedes albopictus*			
*Beilschmiedia erythrophloia*	n.t.	n.t.	---	---
*Beilschmiedia robusta*	n.t.	n.t.	---	---
*Beilschmiedia yunnanensis*	n.t.	n.t.	---	---
*Cryptocarya concinna* (Nam Dong)	30.19 (27.92−33.28)	40.26 (36.49−46.42)	1.922	0.383
*Cryptocarya concinna* (Pu Hoat)	n.t.	n.t.	---	---
*Cryptocarya impressa*	n.t.	n.t.	---	---
*Cryptocarya infectoria*	58.80 (54.40−64.96)	78.50 (71.00−90.93)	1.282	0.527
*Litsea viridis*	n.t.	n.t.	---	---
*Machilus balansae*	n.t.	n.t.	---	---
*Machilus grandifolia*	15.45 (13.62−17.07)	24.47 (22.28−27.88)	3.69 × 10^−5^	1.000
*Neolitsea ellipsoidea*	n.t.	n.t.	---	---
*Phoebe angustifolia*	35.28 (31.29−39.67)	64.97 (57.71−76.08)	23.97	0.000
	*Culex quinquefasciatus*			
*Beilschmiedia erythrophloia*	n.t.	n.t.	---	---
*Beilschmiedia robusta*	n.t.	n.t.	---	---
*Beilschmiedia yunnanensis*	n.t.	n.t.	---	---
*Cryptocarya concinna* (Nam Dong)	41.89 (37.88−46.65)	69.84 (62.41−80.77)	5.550	0.062
*Cryptocarya concinna* (Pu Hoat)	n.t.	n.t.	---	---
*Cryptocarya impressa*	n.t.	n.t.	---	---
*Cryptocarya infectoria*	0.402 (0.000−2.947)	11.39 (0.04−21.64)	6.397	0.041
*Litsea viridis*	n.t.	n.t.	---	---
*Machilus balansae*	n.t.	n.t.	---	---
*Machilus grandifolia*	11.56 (9.13−13.14)	19.24 (17.25−23.07)	0.000	1.000
*Neolitsea ellipsoidea*	4.650 (0.061−7.988)	11.89 (4.63−15.36)	0.002409	0.999
*Phoebe angustifolia*	12.21 (8.66−14.46)	24.28 (21.55−29.24)	0.002467	0.999

n.t. = not tested due to insufficient essential oil.

**Table 7:** Antimicrobial activities of leaf essential oils of Lauraceae from Vietnam.

Sample	Gram (+)			Gram (−)			Yeast
	*Enterococcus faecalis* ATCC 299212	*Staphylococcus aureus* ATCC 25923	*Bacillus* *cereus* ATCC 14579	*Escherichia coli* ATCC 25922	*Pseudomonas* *aeruginosa* ATCC 27853	*Salmonella enterica* ATCC 13076	*Candida albican*s ATCC 10231
	MIC (µg/mL)						
*Beilschmiedia erythrophloia*	32	64	64	n.a.	n.a.	n.a.	128
*Beilschmiedia robusta*	64	64	n.a.	64	n.a.	n.a.	n.a.
*Beilschmiedia yunnanensis*	64	64	64	n.a.	n.a.	n.a.	256
*Cryptocarya concinna* (Pu Hoat)	32	128	64	n.a.	128	256	64
*Cryptocarya impressa*	64	64	128	64	n.a.	n.a.	16
*Cryptocarya infectoria*	128	64	128	n.a.	64	128	64
*Litsea viridis*	16	64	16	n.a.	n.a.	n.a.	128
*Machilus balansae*	64	128	128	n.a.	n.a.	n.a.	n.a.
*Neolitsea ellipsoidea*	16	32	16	128	n.a.	n.a.	128
Streptomycin	256	256	128	32	256	128	n.t.
Nistatin	n.t.	n.t.	n.t.	n.t.	n.t.	n.t.	8
Cyclohexamide	n.t.	n.t.	n.t.	n.t.	n.t.	n.t.	32
	IC_50_ (µg/mL)						
*Beilschmiedia erythrophloia*	10.34	20.34	34.78	n.a.	n.a.	n.a.	56.78
*Beilschmiedia robusta*	20.76	18.67	n.a.	17.88	n.a.	n.a.	n.a.
*Beilschmiedia yunnanensis*	17.99	20.34	24.67	n.a.	n.a.	n.a.	100.34
*Cryptocarya concinna* (Pu Hoat)	8.99	40.67	18.99	n.a.	48.98	145.34	25.67
*Cryptocarya impressa*	20.34	28.77	47.67	18.78	n.a.	n.a.	5.89
*Cryptocarya infectoria*	65.33	32.67	63.56	n.a.	33.22	65.66	32.22
*Litsea viridis*	2.45	18.99	7.67	n.a.	n.a.	n.a.	56.78
*Machilus balansae*	18.78	50.35	45.77	n.a.	n.a.	n.a.	n.a.
*Neolitsea ellipsoidea*	3.99	7.98	5.67	57.78	n.a.	n.a.	56.67

n.a. = not active; n.t. = not tested.

## References

[1] Van der Werff H. (2003). A synopsis of the genus *Beilschmiedia* (Lauraceae) in Madagascar. Adansonia.

[2] Schroeder C.A. (1976). Some useful plants of the botanical family Lauraceae. Calif. Avocado Soc. Yearb..

[3] Krochmal A. (1968). Medicinal plants and Appalachia. Econ. Bot..

[4] Tucker A.O., Maciarello M.J., Burbage P.W., Sturtz G. (1994). Spicebush [*Lindera benzoin* (L.) Blume var. benzoin, Lauraceae]: A tea, spice, and medicine. Econ. Bot..

[5] Rhind J.P. (2020). Essential Oils: A Comprehensive Handbook for Aromatic Therapy.

[6] May P.H., Barata L.E.S. (2004). Rosewood exploitation in the Brazilian Amazon: Options for sustainable production. Econ. Bot..

[7] Chen W., Vermaak I., Viljoen A. (2013). Camphor—A fumigant during the black death and a coveted fragrant wood in ancient Egypt and Babylon: A review. Molecules.

[8] Kong D.-G., Zhao Y., Li G.-H., Chen B.-J., Wang X.-N., Zhou H.-L., Lou H.-X., Ren D.-M., Shen T. (2015). The genus *Litsea* in traditional Chinese medicine: An ethnomedical, phytochemical and pharmacological review. J. Ethnopharmacol..

[9] Missouri Botanical Garden Tropicos. www.tropicos.org.

[10] Beilschmiedia Nees. https://www.discoverlife.org/mp/20q?search=Beilschmiedia.

[11] Salleh W.M.N.H.W., Ahmad F., Yen K.H., Zulkifli R.M. (2015). A review on chemical constituents and biological activities of the genus *Beilschmiedia* (Lauraceae). Trop. J. Pharm. Res..

[12] Wu C.Y., Raven P.H., Hong D.Y. Flora of China. http://www.efloras.org/volume_page.aspx?volume_id=2007&flora_id=2.

[13] Chen J.-J., Kuo W.-L., Sung P.-J., Chen I.-S., Cheng M.-J., Lim Y.-P., Liao H.-R., Chang T.-H., Wei D.-C., Chen J.-Y. (2015). Beilschamide: A new amide, and cytotoxic constituents of *Beilschmiedia erythrophloia*. Chem. Nat. Compd..

[14] Dao N.K. (2017). Flora of Vietnam, Lauraceae Juss.

[15] Yang P.-S., Cheng M.-J., Chen J.-J., Chen I.-S. (2008). Two new endiandric acid analogs, a new benzopyran, and a new benzenoid from the root of *Beilschmiedia erythrophloia*. Helv. Chim. Acta.

[16] Yang P.-S., Cheng M.-J., Peng C.-F., Chen J.-J., Chen I.-S. (2009). Endiandric acid analogues from the roots of *Beilschmiedia erythrophloia*. J. Nat. Prod..

[17] Su Y.-C., Ho C.-L. (2013). Composition and in-vitro cytotoxic activities of the leaf essential oil of *Beilschmiedia erythrophloia* from Taiwan. Nat. Prod. Commun..

[18] Allen C.K. (1942). Studies in the Lauraceae, V: Some eastern Asiatic species of *Beilschmiedia* and related genera. J. Arnold Arbor..

[19] Mabberley D.J. (2008). Mabberley’s Plant-Book.

[20] Chang H.-S., Tang J.-Y., Yen C.-Y., Huang H.-W., Wu C.-Y., Chung Y.-A., Wang H.-R., Chen I.-S., Huang M.-Y., Chang H.-W. (2016). Antiproliferation of *Cryptocarya concinna*-derived cryptocaryone against oral cancer cells involving apoptosis, oxidative stress, and DNA damage. BMC Complement. Altern. Med..

[21] Yang B.-Y., Shi Y.-M., Luo J.-G., Kong L.-Y. (2017). Two new arylalkenyl α,β-unsaturated δ-lactones with cytotoxic activity from the leaves and twigs of *Cryptocarya concinna*. Nat. Prod. Res..

[22] Huang W., Zhang W.-J., Cheng Y.-Q., Jiang R., Wei W., Chen C.-J., Wang G., Jiao R.-H., Tan R.-X., Ge H.-M. (2014). Cytotoxic and antimicrobial flavonoids from *Cryptocarya concinna*. Planta Med..

[23] Kew Science Cryptocarya impressa Miq. http://powo.science.kew.org/taxon/urn:lsid:ipni.org:names:463940-1#source-KBD.

[24] De Kok R.P.J. (2015). A revision of *Cryptocarya* (Lauraceae) from Thailand and Indochina. Gard. Bull. Singapore.

[25] De Kok R.P.J. (2016). A revision of *Cryptocarya* R. Br. (Lauraceae) of Peninsular Malaysia. Kew Bull..

[26] De Kok R.P.J. (2017). Two new records of *Litsea* (Lauraceae) from Singapore and the lectotypification of twenty-two names from several Lauraceae genera. Gard. Bull. Singapore.

[27] Chen Y.-C., Chen I.-S., Guh J.-H. (2007). Cryptocaryone, isolated from *Cryptocarya infectoria*, induces apoptosis through extrinsic pathways: The involvement of death receptor clustering and FADD/caspase-8 activation cascades. Clin. Cancer Res..

[28] Dumontet V., Gaspard C., Van Hung N., Fahy J., Tchertanov L., Sévenet T., Guéritte F. (2001). New cytotoxic flavonoids from *Cryptocarya infectoria*. Tetrahedron.

[29] Othman W.N.N.W., Liew S.Y., Khaw K.Y., Murugaiyah V., Litaudon M., Awang K. (2016). Cholinesterase inhibitory activity of isoquinoline alkaloids from three *Cryptocarya* species (Lauraceae). Bioorganic Med. Chem..

[30] Tang S.-C., Xu W.-B., Wei F.-N. (2010). *Machilus parapauhoi* sp. nov. and a new synonym of *Machilus* (Lauraceae) from east Asia. Nord. J. Bot..

[31] Allen C.K. (1938). Studies in the Lauraceae. I. Chinese and Indo-Chinese species of *Litsea*, *Neolitsea*, and *Actinodaphne*. Ann. Missouri Bot. Gard..

[32] Thang T.D., Dai D.N., Thai T.H., Ogunwande I.A. (2013). Essential oils of *Phoebe angustifolia* Meisn., *Machilus velutina* Champ. ex Benth. and *Neolitsea polycarpa* Liou (Lauraceae) from Vietnam. Rec. Nat. Prod..

[33] Salleh W.M.N.H.W., Ahmad F., Khong H.Y., Zulkifli R.M. (2016). Comparative study of the essential oils of three *Beilschmiedia* species and their biological activities. Int. J. Food Sci. Technol..

[34] Setzer W.N., Haber W.A. (2007). Leaf essential oil composition of five species of *Beilschmiedia* from Monteverde, Costa Rica. Nat. Prod. Commun..

[35] Mondello L. (2016). FFNSC 3.

[36] (2017). NIST17.

[37] Adams R.P. (2007). Identification of Essential Oil Components by Gas Chromatography/Mass Spectrometry.

[38] Yoon W.-J., Moon J.-Y., Kang J.-Y., Kim G.-O., Lee N.H., Hyun C.-G. (2010). *Neolitsea sericea* essential oil attenuates LPS-induced inflammation in RAW 264.7 macrophages by suppressing NF-κB and MAPK activation. Nat. Prod. Commun..

[39] Su Y.-C., Hsu K.-P., Wang E.I.-C., Ho C.-L. (2013). Composition and in vitro anticancer activities of the leaf essential oil of *Neolitsea variabillima* from Taiwan. Nat. Prod. Commun..

[40] Jeong M.-J., Yang J., Choi W.-S., Kim J.-W., Kim S.J., Park M.-J. (2017). Chemical compositions and antioxidant activities of essential oil extracted from *Neolitsea aciculata* (Blume) Koidz leaves. J. Korean Wood Sci. Technol..

[41] Brophy J.J., Goldsack R.J., Fookes C.J.R., Forster P.I. (2002). The leaf oils of the Australian species of *Neolitsea* (Lauraceae). J. Essent. Oil Res..

[42] Padalia R.C., Chanotiya C.S., Thakuri B.C., Mathela C.S. (2007). Germacranolide rich essential oil from *Neolitsea pallens*. Nat. Prod. Commun..

[43] John A.J., Karunakaran V.P., George V., Pradeep N.S., Sethuraman M.G. (2007). Chemical composition and antibacterial activity of leaf oil of *Neolitsea foliosa* (Nees) Gamble var. *caesia* (Meisner) Gamble. J. Essent. Oil Res..

[44] Fontes U.R., Ramos C.S., Serafini M.R., Cavalcanti S.C.H., Alves P.B., Lima G.M., Andrade P.H.S., Bonjardim L.R., Quintans L.J., Araújo A.S. (2012). Evaluation of the lethality of *Porophyllum ruderale* essential oil against *Biomphalaria glabrata*, *Aedes aegypti* and *Artemia salina*. African J. Biotechnol..

[45] Cavalcanti E.S.B., de Morais S.M., Lima M.A.A., Santana E.W.P. (2004). Larvicidal activity of essential oils from Brazilian plants against *Aedes aegypti* L.. Mem. Inst. Oswaldo Cruz.

[46] Govindarajan M. (2010). Chemical composition and larvicidal activity of leaf essential oil from *Clausena anisata* (Willd.) Hook. f. ex Benth (Rutaceae) against three mosquito species. Asian Pac. J. Trop. Med..

[47] Govindarajan M., Benelli G. (2016). Ecotoxicology and environmental safety eco-friendly larvicides from Indian plants: Effectiveness of lavandulyl acetate and bicyclogermacrene on malaria, dengue and Japanese encephalitis mosquito vectors. Ecotoxicol. Environ. Saf..

[48] Hung N.H., Satyal P., Hieu H.V., Chuong N.T.H., Dai D.N., Huong L.T., Tai T.A., Setzer W.N. (2019). Mosquito larvicidal activity of the essential oils of *Erechtites* species growing wild in Vietnam. Insects.

[49] Pavela R. (2015). Acute toxicity and synergistic and antagonistic effects of the aromatic compounds of some essential oils against *Culex quinquefasciatus* Say larvae. Parasitol. Res..

[50] Perumalsamy H., Kim N.-J., Ahn Y.-J. (2009). Larvicidal activity of compounds isolated from *Asarum heterotropoides* against *Culex pipiens pallens*, *Aedes aegypti*, and *Ochlerotatus togoi* (Diptera: Culicidae). J. Med. Entomol..

[51] Ribeiro W.H.F., Vasconcelos J.N., Arriaga A.M.C., de Oliveira M.C.F., Andrade-Neto M., Lemos T.L.G., Santiago G.M.P., Nascimento R.F., Mafezoli J. (2006). *Tephrosia toxicaria* Pers essential oil: Chemical composition and larvicidal activity. Nat. Prod. Commun..

[52] Magalhães L.A.M.I., Lima Mda P., Marques M.O., Facanali R., Pinto A.C., Tadei W.P. (2010). Chemical composition and larvicidal activity against *Aedes aegypti* larvae of essential oils from four *Guarea* species. Molecules.

[53] Santos T.G., Dognini J., Begnini I.M., Rebelo R.A., Verdi M., Dalmarco E.M. (2013). Chemical characterization of essential oils from *Drimys angustifolia* Miers (Winteraceae) and antibacterial activity of their major compounds. J. Braz. Chem. Soc..

[54] Jirovetz L., Bail S., Buchbauer G., Denkova Z., Slavchev A., Stoyanova A., Schmidt E., Geissler M. (2006). Antimicrobial testings, gas chromatographic analysis and olfactory evaluation of an essential oil of hop cones (*Humulus lupulus* L.) from Bavaria and some of its main compounds. Sci. Pharm..

[55] Rather M.A., Dar B.A., Dar M.Y., Wani B.A., Shah W.A., Bhat B.A., Ganai B.A., Bhat K.A., Anand R., Qurishi M.A. (2012). Chemical composition, antioxidant and antibacterial activities of the leaf essential oil of *Juglans regia* L. and its constituents. Phytomedicine.

[56] Schmidt J.M., Noletto J.A., Vogler B., Setzer W.N. (2006). Abaco bush medicine: Chemical composition of the essential oils of four aromatic medicinal plants from Abaco Island, Bahamas. J. Herbs Spices Med. Plants.

[57] Kubo I., Fujita K., Kubo A., Nihei K., Ogura T. (2004). Antibacterial activity of coriander volatile compounds against *Salmonella choleraesuis*. J. Agric. Food Chem..

[58] Liu K., Chen Q., Liu Y., Zhou X., Wang X. (2012). Isolation and biological activities of decanal, linalool, valencene, and octanal from sweet orange oil. J. Food Sci..

[59] Morandim-Giannetti A.A., Pin A.R., Pietro N.A.S., de Oliveira H.C., Mendes-Giannini M.J.S., Alecio A.C., Kato M.J., de Oliveira J.E., Furlan M. (2010). Composition and antifungal activity against *Candida albicans*, *Candida parapsilosis*, *Candida krusei* and *Cryptococcus neoformans* of essential oils from leaves of *Piper* and *Peperomia* species. J. Med. Plants Res..

[60] Venturi C.R., Danielli L.J., Klein F., Apel M.A., Montanha J.A., Bordignon S.A.L., Roehe P.M., Fuentefria A.M., Henriques A.T. (2015). Chemical analysis and in vitro antiviral and antifungal activities of essential oils from *Glechon spathulata* and *Glechon marifolia*. Pharm. Biol..

[61] Ho C.-L., Hsu K.-P., Tseng Y.-H., Wang E.I.-C., Liao P.-C., Chouc J.-C., Linc C.-N., Sua Y.-C. (2011). Composition and antimicrobial activities of the leaf essential oil of *Machilus kusanoi* from Taiwan. Nat. Prod. Commun..

[62] Dai D.N., Chung N.T., Huong L.T., Hung N.H., Chau D.T.M., Yen N.T., Setzer W.N. (2020). Chemical compositions, mosquito larvicidal and antimicrobial activities of essential oils from five species of *Cinnamomum* growing wild in north central Vietnam. Molecules.

[63] Ebadollahi A., Ziaee M., Palla F. (2020). Essential oils extracted from different species of the Lamiaceae plants family as prospective bioagents against several detrimental pests. Molecules.

